# Lower plasma melatonin levels in non-hypoxic premature newborns associated with neonatal pain

**DOI:** 10.1007/s00431-024-05632-1

**Published:** 2024-06-06

**Authors:** Cristina Sánchez-Borja, Delia Cristóbal-Cañadas, María Isabel Rodríguez-Lucenilla, Antonio Muñoz-Hoyos, Ahmad Agil, María Ángeles Vázquez-López, Tesifón Parrón-Carreño, Bruno José Nievas-Soriano, Antonio Bonillo-Perales, Juan Carlos Bonillo-Perales

**Affiliations:** 1 Pediatrics Unit, University Hospital Torrecardenas, Almeria, Spain; 2 Department of Pediatrics, University of Granada, Granada, Spain; 3 Department of Pharmacology, Institute Biohelath & Institute of Neuroscience, University of Granada, Granada, Spain; 4 Nursing, Physiotherapy, and Medicine Department, University of Almería, Ctra. de Sacramento, s/n, La Cañada, Almería, 01410 Spain; 5 Pharmacy Office Holder, Madrid, Spain

**Keywords:** Melatonin, Oxidative stress, Free radicals, Preterm infants, Parenteral nutrition, Neonatal pain

## Abstract

We analyzed plasma melatonin levels in different groups of preterm newborns without hypoxia and their relationship with several perinatal variables like gestational age or neonatal pain. Prospective cohort study of preterm newborns (PTNB) without perinatal hypoxia, Apgar > 6 at 5 min, and oxygen needs on the third day of life. We compared melatonin levels at day 3 of life in different groups of non-hypoxic preterm infants (Student’s *t*-tests, Mann-Whitney *U*, and chi^2^) and analyzed the relationship of melatonin with GA, birth weight, neonatal pain (Premature Infant Pain Profile (PIPP) scale), caffeine treatment, parenteral nutrition, or the development of free radical diseases (correlation study, linear regression) and factors associated with moderate/intense pain and free radical diseases (logistic regression analysis). Sixty-one preterm infants with gestational age (GA) of 30.7 ± 2.0 weeks with no oxygen requirements at day 3 of life were studied with plasma melatonin levels of 33.8 ± 12.01 pg/ml. Preterm infants weighing < 1250 g at birth had lower plasma melatonin levels (*p* = 0.05). Preterm infants with moderate or severe pain (PPIPP > 5) have lower melatonin levels (*p* = 0.01), and being preterm with PIPP > 5 is associated with lower plasma melatonin levels (*p* = 0.03). Being very preterm (GA < 32 GS), having low weight for gestational age (LWGA), receiving caffeine treatment, or requiring parenteral nutrition did not modify melatonin levels in non-hypoxic preterm infants (*p* = NS). Melatonin on day 3 of life in non-hypoxic preterm infants is not associated with later development of free radical diseases (BPD, sepsis, ROP, HIV, NEC).

* Conclusion*: We observed that preterm infants with moderate to severe pain have lower melatonin levels. These findings are relevant because they reinforce the findings of other authors that melatonin supplementation decreases pain and oxidative stress in painful procedures in premature infants. Further studies are needed to evaluate whether melatonin could be used as an analgesic in painful procedures in preterm infants.

* Trial registration*: Trial registration was not required since this was an observational study.

**Table:** 

**What Is Known:** *• Melatonin is a potent antioxidant and free radical scavenger in newborns under stress conditions: hypoxia, acidosis, hypotension, painful procedures, or parenteral nutrition.* *• Pain stimulates the production of melatonin.* *• Various studies conclude that melatonin administration decreases pain during the neonatal period.* **What Is New:** *• Non-hypoxic preterm infants with moderate to severe pain (PIPP>5) have lower levels of melatonin.* *• Administration of caffeine and treatment with parenteral nutrition do not modify melatonin levels in non-hypoxic preterm infants.*

**What Is Known:**

*• Melatonin is a potent antioxidant and free radical scavenger in newborns under stress conditions: hypoxia, acidosis, hypotension, painful procedures, or parenteral nutrition.*

*• Pain stimulates the production of melatonin.*

*• Various studies conclude that melatonin administration decreases pain during the neonatal period.*

**What Is New:**

*• Non-hypoxic preterm infants with moderate to severe pain (PIPP>5) have lower levels of melatonin.*

*• Administration of caffeine and treatment with parenteral nutrition do not modify melatonin levels in non-hypoxic preterm infants.*

## Introduction

Preterm newborns (PTNB) are highly exposed to oxidative stress in their first days of life because they have numerous factors that generate an overproduction of free radicals: transition from intrauterine to postnatal life going from a PO2 of 20–25 mm up to 100 mm Hg, hypoxia in labor or postpartum, occasional needs for mechanical ventilation, acidosis, infections, transfusions, exogenous inputs of free radicals in parenteral nutrition, and exposure to drugs, as well as the numerous painful procedures to which they are exposed in intensive care units. These factors lead to additional production of free radicals that generate oxidative damage [1–7]. Furthermore, newborns, especially premature ones, have a decreased antioxidant capacity [8, 9].

The imbalance between prooxidant factors and the antioxidant capacity of the organism causes tissue damage, leading to “oxygen radical diseases of the newborn”: respiratory distress syndrome (RDS), bronchopulmonary dysplasia (BPD), retinopathy of prematurity (ROP), hypoxic-ischemic disease (HID), intraventricular hemorrhage (IVH), or sepsis and necrotizing enterocolitis (NEC) [1, 4, 10–13]. Melatonin is a potent antioxidant and can effectively scavenge free radicals in newborns [9, 14].

When comparing the antioxidant capacity of melatonin with other antioxidants such as vitamin C, vitamin E, glutathione, and NADPH, melatonin proves to be more efficient in protecting against oxidative stress. One melatonin molecule can eliminate up to 10 reactive oxygen species (ROS) molecules, whereas classical antioxidants can eliminate one or fewer ROS molecules [14, 15]. The antioxidant properties of melatonin make it vital in situations of oxidative stress resulting from unfavorable environmental conditions, such as acidosis and hypotension [16].

During the early stages of pregnancy, the fetus relies on melatonin produced by the mother and placenta, which can cross barriers such as the placenta and blood-brain barrier [17]. The pineal gland produces negligible amounts of melatonin in newborns. Instead, it is primarily produced in organs like the intestine, skin, and bone marrow, where its synthesis and metabolism occur in the mitochondria [18–20].

PTNBs are at a higher risk of experiencing oxidative stress [2, 4] due to their low efficiency in the natural antioxidant system. This stress can result in diseases caused by oxidative damage, especially when the consumption and degradation of melatonin and antioxidants are higher than their synthesis and production capacity [14, 20]. It has been observed that even premature infants who do not have hypoxia can have elevated levels of oxygen radicals in their bodies. However, administering melatonin to such infants can help reduce lipid peroxidation. Studies have shown that melatonin can also help decrease oxidative damage and the frequency of diseases caused by free radicals, such as BPD, HIV, NEC, ROP, and sepsis [9, 10, 12].

Despite these contributions, few studies analyze melatonin blood levels in newborns, especially in PTNB [12, 21, 22]. Furthermore, almost all PTNBs experience perinatal asphyxia and hypoxia and often receive supplemental oxygen, which can significantly alter their melatonin levels [3, 23]. Therefore, we aimed to (1) analyze melatonin levels in PTNB without hypoxia-ischemia or oxygen supplementation; (2) study whether neonatal pain and other perinatal factors can influence circulating melatonin levels; and (3) analyze if these preterm infants without apparent oxidative damage and theoretically “healthy” (of which we do not know other references) develop oxygen radical diseases (BPD, ROP, HIV, NEC or sepsis) and to determine their relationship with neonatal plasma melatonin.

## Materials and methods

### Study design, eligible population, and inclusion criteria

A prospective cohort study was conducted on PTNB under 35 weeks of gestation who were admitted to the Neonatology Unit or Neonatal Intensive Care Unit (NICU) of the Torrecardenas University Hospital in Almeria, Spain. The neonatal ICU of this center provides services to a population of 741,000 people.

The inclusion criteria were preterm newborns < 35 weeks of gestation who did not require supplemental oxygen at 72 h of life and who maintained a SO2 > 92%. The exclusion criteria were (1) perinatal hypoxia, (2) Apgar test score < 7 at 5 min of life, (3) use of supplemental oxygen before 72 h of life with a FiO2 > 0.4, (4) presence of sepsis or arterial hypotension, (5) existence of confirmed metabolic disease, (6) evidence of congenital malformations, (7) need for surgical intervention, (8) diabetic mother, or (9) refusal of the parents to sign the informed consent or to be included in the study.

### Data collection

The care protocols of the Neonatal Unit were adhered to. Therefore, all premature infants under 30 weeks of gestational age and those with apnea pauses received an initial dose of 20 mg/kg of caffeine citrate, followed by a maintenance dose of 5 mg per kilogram every 24 h.

Blood samples were taken on the third day after birth, between 8 and 9 AM, to minimize any impact on the body’s natural melatonin rhythm. None of the selected patients underwent any painful procedures during the 8 h prior to the blood collection.

The variables analyzed were (1) perinatal variables: gestational age at birth (GA), newborn weight (NBW), weight for the gestational age (WGA) [24], and presence of multiple gestation. Before the analytical extraction, the PTNB degree of pain was evaluated using the PIPP scale [25]. The respiratory assistance was also assessed on the third day of life: high flow, CPAP, or BIPAP. (2) Analytical variables: plasma melatonin (Elabscience competitive ELISA kit), human melatonin (MT) with ELISA Kit E-EL-H2016. After centrifugation, the samples were preserved at −20 °C until measurement, lactic acid, hemoglobin, urea, creatinine, GOT, and GPT. (3) Hemodynamic and respiratory variables: mean values of hemodynamic constants taken on the third day of life: heart rate (HR), respiratory rate (RR), and mean arterial blood pressure (MAP).

All preterm infants were followed up to record whether they presented any disease related to oxidative stress of prematurity: sepsis, BPD [26], HIV [27], NEC, and ROP [28].

We compared the different variables between the group of large preterm infants (GA < 32 weeks) and preterm infants 32–34 weeks to evaluate if there were differences between them.

### Statistical study

Frequencies and percentages were used for qualitative variables and means, and standard deviation was used for quantitative variables. The chi-square test (*χ*^2^) was used to compare qualitative variables. The normality or non-normality of the variables was determined using the Kolgomorov-Smirnov test. Student’s *t*-test and the Mann-Whitney *U* test were used to compare the two groups of quantitative variables, and the Kruskal-Wallis test was used for quantitative variables. To compare ordinal variables, we used nonparametric tests. Linear regression analysis was used to evaluate the association of the different variables with melatonin levels, and logistic regression analysis was used to evaluate the variables associated with free radical diseases. All statistical analyses considered *p*-value < 0.05 statistically significant.

### Ethical aspects

The mothers of the newborns gave their written consent before participating in the study. The study followed the Declaration of Helsinki, and the protocol was approved by the Ethics Committee of the Torrecardenas University Hospital, with reference code PI.DCC/MMC-2019.

## Results

Sixty-seven PTNBs met the inclusion criteria. Six patients whose parents did not wish to participate in the study were excluded. Therefore, 61 PTNBs were included in the study. They were between 27 and 34 weeks of gestation, with a mean gestational age (GA) of 30.7 ± 2.0 OS (95% CI, 30.2–31.2 OS), and 29.5% had a GA < 30 OS. A total of 40 preterm infants (65.6%) were from single gestation and 21 (34.4%) from twin gestation. All preterm infants maintained StcO2 > 92% (97.5 ± 1.8%) without any supplemental oxygen supply, although 19 of them (31.1%) required some ventilatory support (4 high flow ventilation and 15 CPAP or BIPAP). All preterm infants had an Apgar at birth at 5 min greater than 6, and 88.5% had an Apgar test at 5 min of life of 9 or 10.

The mean melatonin on the third day of life in preterm infants without perinatal asphyxia and without oxygen needs was 33.8+/−12.01 pg/ml (95% CI, 30.5–37.2 pg/ml), with slightly lower values in preterm infants < 32 weeks without statistically significant differences (*p* = NS) (Table 1). When we compared three gestational age brackets: < 30 weeks (*n* = 18) vs. 30–31 weeks (*n* = 21) vs. 32–34 weeks (*n* = 22), the median melatonin and its minimum and maximum values were respectively 30.1 pg/ml (12.3–59.2 pg/ml) vs. 27.9 og/nk (18.0 pg/ml vs. 51.2 pg/ml) vs. 31.4 pg/ml (21.2–76.6 pg/ml). In the < 32 weeks group, there was a higher incidence of males; they more frequently had moderate or severe pain (PIPP > 5) (*p* = 0.02), more frequently required ventilatory support, were more frequently administered caffeine, had higher parenteral nutrition needs, and had a higher incidence of free radical diseases with low and identical capillary lactic acid levels in both study groups (Tables 1 and 2).

**Table 1:** Characteristics of preterm infants and comparison according to gestational age. Quantitative variables

	All cases	< 32 weeks	32–34 weeks	U*	*p*
	Median (Min., Max.)*	Median (Min., Max.)*	Median (Min., Max.)*		
>*n*	61	39	22		
Melatonin (pg/ml)	30.6 (12.3–76.6)	29.7 (12.3–59.3)	31.4 (21.2–76.6)	334	NS*
Gestational age (weeks)	29.8 (24.1–34.1)	30.2 (24.1–31.6)	32.5 (32.0–34.1)	0.03	< 0.001
Newborn weight (grams)	1350 (800–2055)	1320 (800–2000)	1525 (1025–2055)	291	NS*
Apgar 1 min	8 (2–10)	8 (2–10)	9 (3–10)	322.5	0.1
Apgar 5 min	10 (7–10)	10 (7–10)	9.5 (8–10)	322	NS*
Heart rate	150 (124–194)	148 (130–194)	146 (124–170)	497	NS*
Respiratory rate	53 (25–90)	54 (25–90)	50 (27–69)	494.5	NS*
Mean arterial blood pressure	49 (29.6–77.3)	48 (34.3–66.6)	48.3 (29.6–77.3)	402	NS*
Transcutaneous oxygen saturation (StcO2)	98 (93–100)	98 (94–100)	(98 (93–100)	399	NS*
PIPP score	6 (2–14)	6 (3–14)	5 (2–10)	289	NS*
Lactic acid	1.7 (0.9−3.5)	1.7 (1.1–3.5)	1.7 (0.9−3.0)	360	NS*
Hemoglobin	16.4 (8.4–21.2)	16.1 (10.7–21.2)	17.2 (8.4–20.7)	329.5	NS*

*Min, minimum; *Max*, maximum; *U*, Mann-Whitney *U* test; *NS*, not significant

**Table 2:** Characteristics of preterm infants and comparison according to gestational age. Qualitative variables

	All cases	< 32 weeks	32–34 weeks	RR*	*p*
*n*	61	39	22		
Low weight for gestational age	32.8%	21.6%	52.4%	0.25	0.01
Low intrauterine growth	22%	15.8%	33.3%	0.35	NS*
Multiple gestations	34.4%	23.1%	54.5%	0.25	0.01
Sex male	57.4%	69.2%	36.4%	3.9	0.01
Specify high flow, CPAP, or BiPAP	31.1%	41.0%	13.6%	4.4	0.04
Caffeine administration	43.5%	70.4%	5.3%	42.7	< 0.001
Parenteral nutrition	65.6%	84.6%	31.8%	11.7	< 0.001
PIPP score > 5	50.8%	61.5%	31.8%	3.4	0.02
Bronchopulmonary dysplasia	8.3%	13.2%	0%	–	–
Retinopathy of prematurity	3.3%	5.3%	0%	–	–
HIV	18.3%	26.3%	4.5%	7.5	0.04**
Necrotizing enterocolitis	3.3%	5.5%	0%	–	–
Sepsis	18.3%	26.3%	4.5%	7.5	0.04
Oxidative stress disease	23.7%	35.1%	4.5%	11.3	0.01

*RR, relative risk (chi^2^); *NS*, not significant; **Fisher’s exact test

**Fig. 1:**
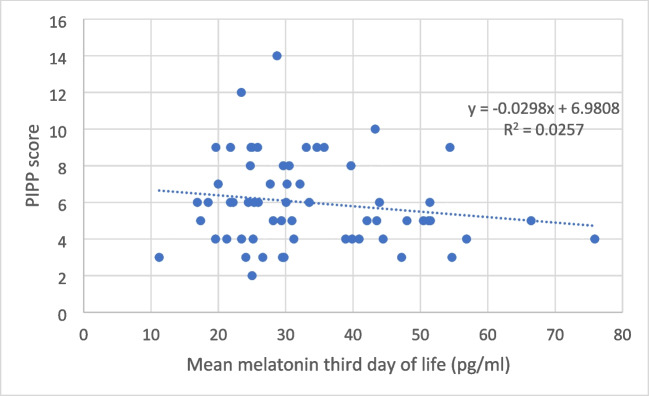
Correlation between melatonin levels and PIPP scale values

Preterm infants weighing < 1000 g (*p* = 0.002), preterm infants < 1250 g (*p* = 0.05), male preterm infants (*p* = 0.04), and preterm infants with moderate to severe pain (PIPP score > 5) (*p* = 0.01) had lower melatonin levels (Table 3). Figure 1 depicts the relationship between melatonin and the PIPP score.

**Table 3:** Differences in plasma melatonin according to different risk factors

	*n*	With risk factor	No risk factor	*U*	*p*
		Median (Min., Max.)*	Median (Min., Max.)*		
		Melatonin (pg/ml)	Melatonin (pg/ml)		
Multiple gestations	21	31.6 (18.5–76.6)	29.6 (12.3–59.3)	480	NS
Low weight for gestational age	19	30.6 (18.0–49.8)	30.5 (18.1–49.7)	381.5	NS
Low intrauterine growth	13	29.5 (18.0–45.1)	31.3 (12.3–76.6)	263	0.08
Gestational age < 32 weeks	39	29.8 (12.3–59.3)	31.4 (21.2–76.6)	334	NS
Gestational age < 30 weeks	18	30.1 (12.3–59.3)	30.6 (18.1–76.6)	362.5	NS
Birth weight < 1500 g	41	30.5 (12.3–54.6)	30.9 (18.5–78.6)	103.5	0.07
Birth weight < 1250 g	22	30.0 (12.3–55.2)	30.7 (18.3–77.6)	98.5	0.05
Birth weight < 1000 g	7	25.9 (19.9–34.6)	31.0 (12.3–76.6)	115.5	0.09
Male sex	35	33.1 (12.3–76.6)	27.9 (18.0–54.6)	345.5	0.04
Required high flow ventilation, CPAP, or BIPAP	19	30.2 (12.3–54.6)	31.0 (18.0–76.6)	369	NS
Caffeine administration	20	27.9 (12.3–54.6)	31.8 (21.2–76.6)	172.5	0.08
Parenteral nutrition administration	40	31.8 (21.2–76.6)	29.7 (12.3–76.6)	496	NS
PIPP score > 5	31	27.1 (18.3–54.6)	35.6 (12.3–76.6)	330	0.03
Bronchopulmonary dysplasia	5	25.9 (19.9–54.6)	30.6 (12.3–76.6)	125.5	NS
Retinopathy of prematurity	2	49.3 (44.1–54.6)	30.4 (12.3–76.6)	102	0.07
HIV	11	30.6 (12.3–50.4)	30.5 (18.0–76.6)	272.5	NS
Necrotizing enterocolitis	2	37.4 (24.4–50.4)	30.5 (12.3–766.6)	64	NS
Sepsis	11	30.7 (12.3–54.6)	30.5 (12.3–76.6)	254	NS
Oxidative stress disease	14	30.6 (12.3–54.6)	30.5 (18–76.6), 0	322	NS

*Min, minimum; *Max*, maximum; *U*, Mann-Whitney *U* test; *NS*, not significant

Moderate or severe pain (PIPP score > 5) was found in 50.8% of preterm infants without asphyxia and oxygen needs. Presenting a PIPP score > 5 was associated with lower plasma melatonin levels (*p* = 0.03) (Table 4). Receiving caffeine treatment or parenteral nutrition was not associated with different plasma melatonin levels (*p* = NS), as seen in Tables 3 and 4.

**Table 4:** Factors associated with plasma melatonin levels or free radical disease in preterm without hypoxia

	Factors associated with plasma melatonin (1)*			Factors associated with free radical disease (2)*		
	Beta	CI 95% *	*p*	OR*	CI 95%*	*p*
Melatonin (pg/ml)	–	–	–	0.9	0.9–1.0	NS*
Multiple gestations	0.1	−2.6–11.3	NS*	0.9	0.9–1.0	NS*
Low weight for gestational age	−0.06	−8.9–5.3	NS*	2.5	0.7–8.6	NS*
Low intrauterine growth	−0.1	−12.7–3.8	NS*	3.8	1.01–14.6	0.04
Gestational age	0.7	−0.9–2.3	NS*	0.7	0.5–1.0	NS*
Gestational age < 30 weeks	−3.8	−10.8–3.0	0.26	11.3	1.3–94.4	0.02
Gestational age < 30 weeks	−1.3	−8.6–6.0	NS*	2.6	0.7–9.3	NS*
Birth weight	0.01	−0.01–0.02	0.07	0.9	0.9–0.9	0.03
Birth weight < 1500 g	−6.1	−13.1–0.8	0.08	2.2	0.5–9.1	NS*
Birth weight < 1250 g	−5.1	−12.0–1.7	0.10	3.2	0.9–11.3	0.06
Birth weight < 1000 g	−8.6	−18.9–1.6	0.09	3.8	0.6–21.5	0.10
Male sex	−5.8	−12.8–0.8	0.08	2.3	0.6–8.7	NS*
Apgar 1 min	0.3	−1.4–2.0	NS*	0.7	0.5–0.9	0.02
Apgar 5 min	0.1	−4.0–4.2	NS*	0.4	0.2–1.0	0.05
Heart rate	0.03	−0.2–2.9	NS*	1.9	0.9–1.0	NS*
Respiratory rate	−0.03	−0.2–0.2	NS*	0.9	0.9–1.0	NS*
Mean blood pressure	0.07	−0.4–0.4	NS*	0.9	0.9–1.0	NS*
Oxygen saturation (StcO2)	0.4	−1.3–2.3	NS*	1.1	0.8–1.5	NS*
Assisted ventilation required	−2.9	−10.1–4.3	NS*	5.3	1.4–19.2	0.01
PIPP score	−0.8	−2.1–0.5	NS*	1.1	0.8–1.4	NS*
PIPP score > 5	−7.1	−13.5–10.6	0.03	2.25	0.6–7.7	NS*
Lactic acid	−1.9	−7.5–3.7	NS*	0.8	0.3–2.4	NS*
Caffeine administration	−5.7	−13.6–2.1	0.14	3.5	0.8–14.5	0.08
Use of parenteral nutrition	3.7	−3.7–10.7	NS*	1.5	0.4–5.6	NS*

*(1), linear regression analysis; *(2)*, logistic regression analysis; *CI*, confidence interval; *OR*, odds ratio; *NS*, not significant

23.7% of preterm infants between 27 and 34 OS without perinatal asphyxia and without oxygen needs presented some free radical disease (*n* = 14) (Table 4), 5 cases presented BPD, 11 cases of HIV (72% grade I), 11 cases clinical late sepsis, 2 cases NEC, and 2 ROP. We did not observe an association between plasma melatonin levels on the third day of life and the subsequent development of free radical disease. Table 4 describes the factors associated with the development of free radical disease.

## Discussion

It is difficult to precisely determine the production capacity and circulating levels of melatonin in PTNB due to the need for studies on this group of children [12, 21, 22]. Melatonin levels can vary significantly due to factors that increase oxidative stress in PTEN. These factors include episodes of pain that increase free radicals, the requirement for supplemental oxygen, and an imbalance between melatonin consumption and production [3, 10, 14, 18, 29].

In our study, we found that preterm infants aged 27–34 weeks (GA, 30.7+/−2 weeks) on the third day of life had plasma melatonin levels of 33.8 ± 12.01 pg/ml (95% CI, 30.5–37.2 pg/ml). Our results agree with those observed by Marseglia et al. [12]. Table 1 shows that plasma melatonin levels are practically identical in preterm infants < 30 weeks and those born between 31 and 32 weeks. However, preterm infants < 32 weeks have lower plasma melatonin levels than those born between 32 and 34 weeks. Other authors have reported lower melatonin levels in preterm infants, with a more significant relationship between lower gestational age and melatonin levels in larger infants [21, 30].

The lower association of melatonin with gestational age and higher plasma melatonin levels in our study may be due to two aspects: (1) Our premature infants who did not have perinatal asphyxia and did not require oxygen had lower levels of oxidative stress and consumed less melatonin [1, 2, 10, 12, 14]. This aspect suggests that few preterm infants in our study had deficient melatonin levels. 9.8% had plasma melatonin levels < 10 pg/ml, whereas in the prospective multicenter study by Biran et al., 81% of their preterm infants < 34 weeks had plasma melatonin < 7 pg/ml on the third day of life. (2) In our study, there were very few preterm infants < 28 weeks or weighing < 1000 g (*n* = 7, 11.4%), which were those with the most significant oxidative damage and consequently would have a higher melatonin consumption [1, 10–12].

Concerning birth weight, we observed that preterm infants under 1250 g had lower melatonin levels than those over 1250 g (*p* = 0.05), and preterm infants < 1500 g have slightly lower melatonin levels (*p* = 0.06) (Table 2) with a decrease of 6.1 pg/ml of their melatonin levels (0.08). At lower weights, there were usually lower melatonin levels (*r* = 0.22, *p* = 0.07), as shown in Tables 3 and 4. Muñoz-Hoyos et al. [22] analyzed plasma melatonin levels in newborns with respiratory distress. They described newborns < 1500 g had significantly lower levels, probably because their study included term newborns, who usually have higher melatonin levels than PTNBs [31].

Preterm infants in the intensive care unit (ICU) undergo numerous painful procedures, increasing their oxidative stress levels (6.36). Although pain stimulates melatonin production in a physiological attempt to eliminate the free radicals generated, melatonin is often consumed to neutralize them [1, 32, 33]. Moderate or severe pain (PPIPP > 5) is present in 50.8% of preterm infants without asphyxia and oxygen needs, being especially frequent in preterm infants < 32 weeks (Table 2). We have observed that preterm infants with moderate–severe pain (PIPP > 5) have lower plasma melatonin levels (*p* = 0.01) (Table 3) and being preterm with PIPP > 5 was associated with lower plasma melatonin levels (*p* = 0.03) (Table 4).

The low levels of melatonin in PTNB with moderate to severe pain may be due to an imbalance between melatonin production and consumption. This imbalance results in higher consumption that cannot be counteracted by the immature antioxidant system of these children [6, 34]. This consideration may explain why melatonin administration has been shown to decrease pain and oxidative stress markers during painful procedures. Melatonin administration has also been observed to decrease the PIPP score in preterm infants [7, 33].

It has been reported that certain drugs, like caffeine, can elevate melatonin levels in the blood by competing for the same metabolic pathway [35]. Caffeine treatment was received by 43.5% of our preterm infants, and no differences in melatonin levels were observed between preterm infants who received caffeine and those who did not, likely due to the significant individual variability in the bioavailability of this drug and the hepatic metabolism in PTNB [29].

Parenteral nutrition is an exogenous source of free radicals, where oxidative stress can come from in vitro nutrient oxidation of solutions and in vivo reactions when intravenous prooxidant molecules are infused [5, 36]. Our study found that receiving parenteral nutrition did not impact melatonin levels in preterm infants who did not experience hypoxia. This finding may be explained because these infants are less exposed to free radicals, and their natural melatonin production is sufficient to counteract any exogenous free radicals. Additionally, protecting parenteral nutrition from light and adding mono- and polyunsaturated fatty acids (PUFA) minimizes free radical formation [37].

Low melatonin levels are one of the determinant factors in the development of free radical diseases (BPD, ROP, HIV, NEC, and sepsis) in PTNB [16, 30]. In preterm infants without hypoxia, free radical diseases also occur in 23.7% of cases [37]. We observed that most preterm infants can produce melatonin levels above 25 pg/ml at 72 h of life [12]. On the third day of life, there were no differences between the melatonin levels at third day of life of preterm infants who developed free radical disease (sepsis, ROP, BPD, HIV, or NEC) probably because many factors associated with free radical disease occurred after the third-day melatonin was measured (late sepsis in 18% of cases, enterocolitis, etc.). Melatonin was not associated with developing these diseases. These diseases were associated with lower gestational age, lower birth weight, lower Apgar test score at birth, and requiring mechanical ventilation or moderate–severe pain on the third day of life, as shown in Tables 3 and 4 [1, 8, 9, 37].

Deficiency of melatonin at the mitochondrial level may be the primary cause of free radical diseases, rather than plasma melatonin levels, as melatonin synthesis and metabolization occur significantly at the mitochondria [18–20]. Mitochondria contain much higher concentrations of melatonin in their granules compared to the melatonin levels found in the bloodstream [38]. It has been demonstrated that free radicals at the mitochondrial level can cause cellular injury implicated in the pathogenesis of free radical diseases due to oxidative self-injury and mitochondrial dysfunction [39, 40]. This reasoning is supported by the fact that premature infants treated with melatonin have a decreased incidence of free radical diseases [10, 12, 30, 41] when plasma melatonin levels 500–1000 times higher than physiological levels are reached. Since melatonin has a great facility to cross cell membranes [17], these doses may increase melatonin at the mitochondrial level, which could explain the clinical improvement and prevention of free radical disease seen with its administration.

Ongoing clinical trials, such as the one registered on Clinical Trial.gov (NCT04235773) [42], will help us better understand the effects of administering melatonin to preterm infants below 30 weeks of age. These trials will evaluate the pharmacokinetic and metabolic properties of melatonin at these ages and its effectiveness in preventing free radical diseases that commonly cause neurological morbidity and high mortality [43–46].

The study has some strengths: it followed a prospective longitudinal design, and the pain scale assessment was performed exclusively by two trained nurses, which reduced inter-subject variability. However, some limitations also exist. The most relevant is the presence of other factors that may affect melatonin levels. These factors include light stress due to light intensity in the neonatal ICU, acoustic stress, emotional stress from the kangaroo program, antibiotic treatment, free radicals present in parenteral nutrition, or other factors not accounted for in this study.

## Conclusions

We observed that preterm infants with moderate to severe pain have lower melatonin levels. These findings are relevant because they reinforce the findings of other authors that melatonin supplementation decreases pain and oxidative stress in painful procedures in premature infants. To reduce the damage caused by oxidative stress, we should avoid unnecessary extractions and minimize pain in premature infants. Further studies are needed to evaluate whether melatonin could be used as an analgesic in painful procedures in preterm infants.

## Data Availability

The data that support the findings of this study are not openly available due to reasons of sensitivity and are available from the corresponding author upon reasonable request.

## Abbreviations

BDP: Bronchopulmonary dysplasia
GA: Gestational age at birth
HID: Hypoxic-ischemic disease
IVH: Intraventricular hemorrhage
MAP: Arterial blood pressure
MT: Human melatonin
NEC: Necrotizing enterocolitis
NICU: Neonatology Unit or Neonatal Intensive Care Unit
PIPP: Premature Infant Pain Profile
PTNB: Preterm newborns
RDS: Respiratory distress syndrome
ROP: Retinopathy of prematurity
ROS: Reactive oxygen species
WGA: Weight for the gestational age
